# A Narrative Review of Existing Options for COVID-19-Specific Treatments

**DOI:** 10.1155/2021/8554192

**Published:** 2021-11-12

**Authors:** Wasey Ali Yadullahi Mir, Abdul Hasan Siddiqui, Gautam Valecha, Shawn Patel, Fatima Ayub, Riddhi Upadhyay, Sana Ahmed Alhajri, Suman Gaire, Dhan Bahadur Shrestha

**Affiliations:** ^1^Department of Internal Medicine, Mount Sinai Hospital, Chicago, IL, USA; ^2^Department of Oncology, Presbyterian Healthcare Services, Albuquerque, NM, USA; ^3^Department of Internal Medicine, The Carle Illinois College of Medicine, Champaign, IL, USA; ^4^Department of Pediatrics, University of Illinois Chicago, Chicago, IL, USA; ^5^Department of Emergency Medicine, Palpa Hospital, Palpa, Nepal

## Abstract

The new coronavirus disease 2019 (COVID-19) was declared a global pandemic in early 2020. The ongoing COVID-19 pandemic has affected morbidity and mortality tremendously. Even though multiple drugs are being used throughout the world since the advent of COVID-19, only limited treatment options are available for COVID-19. Therefore, drugs targeting various pathologic aspects of the disease are being explored. Multiple studies have been published to demonstrate their clinical efficacy until now. Based on the current evidence to date, we summarized the mechanism, roles, and side effects of all existing treatment options to target this potentially fatal virus.

## 1. Introduction

Coronavirus disease 2019 (COVID-19), caused by severe acute respiratory syndrome coronavirus 2 (SARS-CoV-2), was declared a global pandemic in early 2020. As of July 19, 2020, COVID-19 has claimed around four million lives worldwide, with total cases of more than 189 million. The United States alone corresponds to more than 33 million cases around the globe [1].

COVID-19 continues to impact many countries globally. Primary treatment continues to be centered on alleviating symptoms and managing the complications of COVID-19. Since the advent of COVID-19, several drugs and drug combinations have been studied [2]. Many of these treatments are based on the in vitro activity against SARS-CoV and MERS-CoV. However, diligent work in finding an effective treatment is still in the process, including observational studies, randomized controlled trials, and case series. There are limited evidence and data to support any effective and gold standard therapy against COVID-19. It is a general understanding that most of the patients who have minimal to mild symptoms of COVID-19 do not require any COVID-19-specific treatment, and therefore, supportive measures are usually sufficient. For example, we rely on quarantine, isolation, and infection control measures to prevent disease spread and supportive care for those who become ill. However, patients with moderate to severe symptoms, including subjective dyspnea, hypoxia, recurrent fever, or signs of organ failure, may be candidates for COVID-19-specific therapy.

## 2. Methods

We searched the PubMed, EMBASE, and Google Scholar for all articles related to COVID-19-specific therapies using the search words, COVID-19, SARS-CoV-2, Coronavirus, and search keyword for some of the most common medicines, including chloroquine, hydroxychloroquine, remdesivir, tocilizumab, leronlimab, convalescent plasma, azithromycin, lopinavir-ritonavir, ribavirin, favipiravir, oseltamivir, and umifenovir.

## 3. Results and Discussion

The treatments reviewed in this summary can be divided into five categories: immunomodulatory drugs, antiviral, COVID-19, specific immunoglobulins, convalescent plasma, and various drugs (Table 1).

### 3.1. Immunomodulatory Drugs

Severe acute respiratory syndrome coronavirus 2 (SARS-CoV-2) may cause severe illness in up to 20% of patients, which can be attributed to a systemic hyperinflammatory response.

Therefore, de-escalation of this response is a potential therapeutic target. Some of the immunomodulators studied and recommended for the treatment of COVID-19 will be discussed in this section.

#### 3.1.1. Chloroquine and Hydroxychloroquine

Chloroquine (CQ) and hydroxychloroquine (HCQ) emerged earlier in the pandemic and have gained tremendous public attention. On March 30th, 2020, the U.S. Food and Drug Administration (US FDA) issued an emergency use authorization (EUA) for CQ/HCQ to treat hospitalized patients with COVID-19 [3]. Guidelines were formulated for this drug to be administered to hospitalized patients who had evidence of pneumonia, and it continues to be widely used in acute COVID-19 worldwide [4].

CQ and HCQ have been long used to treat and prevent malaria in many malaria-endemic countries. Apart from malaria, HCQ is also the cornerstone of the treatment of several autoimmune and inflammatory disorders. There is convincing evidence from in vitro studies that HCQ has antiviral activity against SARS-CoV-2 [5]. It has antiviral potential through multiple mechanisms, mainly through interference with the terminal glycosylation of the cellular receptor ACE2, thereby preventing virus-receptor binding and entry into cell [6]. Secondly, HCQ increases the pH of acidic cellular organelles, hampering various viral proteins [7]. HCQ has an immunomodulatory effect, which could be explained by its propensity to alter intracellular pH leading to the inhibition of lysosomal activity in antigen-presenting cells and B cells, thereby preventing T-cell activation and cytokine production, respectively [7]. The other possible immunomodulatory action is partly mediated by inhibiting tumor necrosis factor (TNF) release from the monocytes and macrophages that play a significant role in activating other immune cells apart from starting the endothelial cells [8–10].

In one of the earliest reports from China containing 100 patients from 10 hospitals, HCQ was associated with improved radiologic findings, enhanced viral clearance, and decreased disease progression [11]. In an open-label, nonrandomized French study by Gautret et al. consisting of 36 COVID-19-positive patients, the group treated with 200 mg of HCQ (*n* = 20) had improved virologic clearance than the control group (*n* = 16) with standard treatment. The virological clearance measured by nasopharyngeal swab was 70% in the HCQ group versus 12.5% in the control group (*P*=0.001). Moreover, the addition of azithromycin (AZT) to HCQ in 6 patients was associated with improved viral clearance (6/6, 100%) compared with HCQ monotherapy (8/14, 57%). However, this study had several significant limitations, including a small sample size and not reporting clinical or safety outcomes [12]. In another study by Gautret et al., 80 mildly symptomatic COVID-19 patients received HCQ 200 mg for ten days along with AZT 500 mg for one day, followed by 250 mg for four days [13]. They observed significant clinical improvement and viral clearance. However, this study's major drawback is that it was an observational single-arm study. Therefore, its clinical efficacy and safety cannot be extrapolated to patients with severe disease because this was done in mild diseases. An observational study in China consisting of 30 patients randomized to HCQ 400 mg daily for five days along with the standard of care versus just standard care alone reported no significant difference in viral clearance between the two groups (86.7% vs. 93.3%; *P* > 0.05) or median duration of viral clearance (4 days vs. two days; *P* > 0.05) [14]. Geleris et al., in a study consisting of 1376 patients, reported a primary endpoint of intubation or death in 25.1% of patients with no added risk or benefit of HCQ administration [15]. In contrast, Arshad et al. showed that in patients with COVID-19 treated with HCQ alone and combined with AZT, it was correlated with a decline in COVID-19-associated mortality [16].

A study among 368 COVID-19 patients in which 97 received HCQ alone, 113 received HCQ and AZT, and 158 were unexposed to HCQ resulted in a higher risk of death in patients treated with HCQ alone, without any difference between these groups for the risk of ventilation [17]. In a study of 1438 patients, HCQ use was not associated with decreased in-hospital mortality, whether associated with AZT or used alone [18]. In a survey from Brazil among mild-to-moderate COVID-19 cases, the use of HCQ alone (odds ratio, 1.21; 95% confidence interval [CI], 0.69 to 2.11; *P*=1.00) or with AZT (odds ratio, 0.99; 95% CI, 0.57 to 1.73; *P*=1.00), did not improve clinical status at 15 days as compared with standard care [19]. Prolongation of the corrected QT interval and elevated liver-enzyme levels were more frequent in patients receiving HCQ, alone or with AZT [19]. In a randomized clinical trial of 75 patients treated with HCQ with a higher dose loading dose of 1200 mg/day for three days followed by 800 mg/day for 2 or 3 weeks, compared with 75 patients receiving standard of care alone, there was no difference in viral clearance at day 28 and no clinical difference between the two groups [20]. A recent study in ambulatory patients with mild symptoms also indicated that HCQ did not reduce symptom severity at 14 days [21]. Similarly, Mitjà et al. reported no benefit of HCQ in patients with mild COVID-19 beyond routine care [22].

Boulware et al. studied the postexposure prophylactic role of HCQ. They found no significant benefit of HCQ in the incidence of new illness (49 of 414 [11.8%]) for those receiving placebo (58 of 407 [14.3%]) [23]. HCQ could not prevent the occurrence of COVID-19 after moderate or high exposure to COVID-19 [23]. In contrast, in a similar study in healthcare workers, consumption of four or more maintenance doses of HCQ was associated with a significant decline in the odds of getting infected (AOR: 0.44; 95% CI: 0.22–0.88), leaving the question of utility of HCQ postexposure prophylaxis open in that high-risk population [24].

CQ and HCQ, due to their inhibitory effect on potassium channels (IKr current), increase the risk of several arrhythmias and EKG changes [25]. This side effect is dose-dependent; however, serious arrhythmias have been reported even at therapeutic doses. These severe cardiac side effects, including QT prolongation, Torsade de Pointes, and arrhythmia, were reported in many studies [18, 26–29]. In a randomized trial, adverse events were observed in about 30% of patients who received HCQ compared with only 9% of patients in a control group [20]. A QTc >500 ms was observed in 11% to 20% of patients receiving HCQ and AZT [30, 31]. This was frequently seen with a high dose of HCQ (1200 mg/day for ten days) combined with AZT [32]. Among 40 patients with severe infection (admitted to intensive care units) who received either HCQ or HCQ + AZT, there was an increase in QTc in 37 of 40 patients (93%) after drug administration [33]. Besides cardiotoxicity, HCQ also increases the risk of retinopathy, acute pancreatitis, neutropenia, hepatotoxicity, and anaphylaxis [28, 34].

Both CQ and HCQ have clinically significant drug interactions. AZT is a macrolide that may prolong the QT/QTc interval leading to a higher risk of cardiac death [35, 36]. The FDA has warned that AZT can lead to potentially fatal arrhythmias and discourages the use of this antibiotic among patients with underlying heart disease and those with known electrolyte imbalance [37]. Therefore, CQ or HCQ and AZT's concomitant use potentially places patients at a higher risk of cardiotoxicity. Other treatments used in managing COVID-19 patients may show drug interaction with CQ/HCQ. Lopinavir/ritonavir strongly inhibits CYP3A4 and has a large number of significant drug interaction concerns [38]. In in vitro studies, remdesivir appears to be a substrate for the drug-metabolizing enzymes CYP2C8, CYP2D6, and CYP3A4 [39]. Therefore, significant potential drug interactions need to be acknowledged when selecting a treatment.

Taken in totality, the available evidence is limited, and most studies are hampered by limited sample size and study design. Furthermore, meta-analysis does not suggest any efficacy of HCQ in patients with COVID-19 [40]. Early results from in vitro studies and uncontrolled case studies created a massive hype for HCQ, aggravated by media and social excitement, which caused a massive demand despite limited data.

#### 3.1.2. Corticosteroids

Although steroids are usually considered an essential treatment modality in inflammatory conditions, their role in COVID-19 remains debatable. One of the frequently observed causes of mortality is hemophagocytic lymphohistiocytosis (sHLH), another hypercytokinemia syndrome associated with multiorgan failure, thought to be precipitated COVID-19 [41]. Evidence from the limited literature suggests an increased production of cytokines leading to the activation of other inflammatory cells and endothelium activation, resulting in multiorgan damage and failure. The most crucial role of corticosteroids is reducing the exudative fluid in lung tissue, thereby improving hypoxia and minimizing the risk of respiratory failure [42]. Corticosteroids were increasingly used worldwide in as much as 50% of patients affected by COVID-19 particularly in China [39, 43]. The few retrospective studies earlier in the pandemic reported inconclusive results, including patients with severe COVID-19 patients with pulmonary involvement [44–48]. Among the five studies (4 retrospective and one quasiprospective study), three studies have shown a benefit, and the other two studies reported no use. Complicating the decision making, one study observed significant harm, especially in critical and severe cases (propensity-matched adjusted hazard ratio [HR] 2.90; 95% CI, 1.17–7.16; *P*=0.021) [44–48]. Taken as a whole, the studies suggest that corticosteroids improve the condition of severe and critically ill COVID-19 patients in many ways, including decreased duration of hospital stay, improvement in the status of oxygenation, the reduced requirement to intubate and subsequent ventilation, prevention of ventilator parameters worsening, progression to ARDS, and death [46–48].

A large prospective study conducted in the UK (The RECOVERY Trial) randomized 2104 patients to dexamethasone 6 mg per day (oral or intravenous) for ten days, with 4321 patients receiving usual care [43]. Patients included in this study had various comorbidities, including diabetes (24%), heart disease (27%), and chronic lung disease (21%), and 56% had at least one major comorbidity. In this study, 54% of patients were below 70 years, and 22% were between 70 and 80 years. The primary endpoint was mortality within 28 days, which was significantly less in the dexamethasone group compared with the routine care group (21.6% vs. 24.6%; age-adjusted rate ratio [RR], 0.83; 95% CI, 0.74–0.92; *P* < 0.001). No benefit was observed in mild or moderate cases without oxygen requirement in mortality reduction at the end of 28 days (17.0% vs.13.2%; RR, 1.22; 95% CI, 0.93–1.61; *P*=0.14). However, there was 35% reduction in mortality in intubated patients (29.0% vs. 40.7%; RR, 0.65; 95% CI, 0.48–0.88; *P*=0.003) and 20% reduction among the patients on supplemental oxygen therapy (21.5% vs. 25.0%; RR, 0.80; 95% CI, 0.67–0.96; *P*=0.0021). This study also reported a shorter duration of hospital stay with dexamethasone compared with standard routine care (median 12 days vs. 13 days) and a higher probability of discharge within 28 days with dexamethasone (11%; RR, 1.11; 95% CI, 1.04–1.19; *P*=0.002) [49].

The most frequently used corticosteroids are methylprednisolone and dexamethasone, secondary to their high bioavailability in the lungs. Some studies have reported that corticosteroid usage is associated with delayed viral clearance in patients with a previous viral illness; the same results were reported in COVID-19 patients [50–52]. Zhou et al. reported delayed clearance of the virus and increased mortality risk with the early administration of corticosteroid in COVID-19 patients [53]. However, a short course of corticosteroids in some studies was beneficial in this regard [54]. Furthermore, corticosteroid use could predispose patients to secondary bacterial infection and various electrolytes derangements, including hypokalemia hyperglycemia [54]. However, there is inconsistent data to show any superiority among dexamethasone or methylprednisone use in COVID-19 [55, 56].

Although the data from retrospective studies do not strongly support corticosteroid use in COVID-19, the RECOVERY trial, in particular, indicates a beneficent role in reducing mortality and duration of hospital stay among moderate to moderately severe cases.

#### 3.1.3. Tocilizumab

Tocilizumab is a recombinant humanized monoclonal antibody targeted against the IL-6 receptor. It is approved for the treatment of various autoimmune and inflammatory disorders, including rheumatoid arthritis [57]. A retrospective study by Ruan et al. reported higher IL-6 levels associated with disease progression and fatal outcome [58]. The role of IL-6 in mediating the inflammatory response in COVID-19 is a vital target in halting the marked inflammatory response. In critically ill COVID-19 patients who have elevated levels of IL 6, the efficacy of tocilizumab has been reported by retrospective studies [59]. 20 critically ill patients who received tocilizumab, there was a marked improvement in fever and other clinical symptoms [60]. Moreover, 15 of 20 required less oxygen, and there was an overall improvement in the CT scans in 19 patients. There were no significant adverse reactions observed. Of the 21 patients, 20 fully recovered after tocilizumab treatment and were discharged within two weeks [60]. A retrospective study in fifteen critically ill patients evaluated the efficacy and safety of tocilizumab as a monotherapy or in combination with methylprednisolone. There was a clinical improvement and a decrease in IL-6 and C-reactive protein (CRP) levels [59].

Another phase III clinical trial approved by the FDA evaluates tocilizumab in hospitalized patients with severe COVID-19 pneumonia [NCT04320615]. . Notably, in the latest trial of tocilizumab (COVACTA trial) among 452 patients, tocilizumab (*n* = 294) did not meet the primary endpoint of improved clinical status after seven days of administration (*P*=0.36) compared with the placebo group (*n* = 144). Moreover, there was no difference in mortality at day 28 between tocilizumab (19.7%) and placebo (19.4%) (0.3% [95% CI, −7.6 to 8.2]; *P*=0.94) [61]. In a randomized, controlled, open-label trial including 4116 patients (RECOVERY), there was a decrease in 28 days mortality in the tocilizumab arm (rate ratio 0·86; 95% confidence interval [CI] 0·77–0·96; *P*=0.007). Furthermore, tocilizumab also decreased the chances of mechanical ventilation and death in those patients who were not ventilated. The mortality benefits of tocilizumab were seen in patients taking steroids [62]. However, there is a need for more robust studies to identify the role of tocilizumab in reducing mortality benefits for COVID-19 patients.

#### 3.1.4. Interferons

Interferons are a broad spectrum of immunomodulators with antiviral properties. IFN-*α* has been used to treat coronavirus diseases previously, such as SARS and MERS [63, 64]. In addition, interferon -*α* (IFN*α*) and −*β* (IFN*β*) have been recommended for the treatment of COVID-19 [65–67].

Interferons bind to its receptor on the cell membrane and then phosphorylate STAT1 and other transcription factors. STAT1 translocates to the nucleus, leading to interferon-stimulated genes (ISGs), which mediates the immunomodulatory effects and interferes with viral replication [67]. IFN*α* and IFN*β* are frequently studied as a combination therapy with ribavirin and or lopinavir-ritonavir [29, 68–70]. IFN*β* has superior efficacy against coronaviruses compared to IFN*α* [70–72]. Furthermore, some *in vitro* studies indicate that IFN*β* induces anti-inflammatory adenosine secretion and maintains endothelial barriers when used in early stages of infection [67]. IFN*α*, in combination with ribavirin, has been recommended for COVID-19 patients in China [72]. SARS-CoV-2 is more sensitive to prophylactic IFN-1 administration [67, 70–72]. *In vitro* pretreatment studies with INF-1 have confirmed this [73]. Due to limited evidence, routine usage cannot be recommended until further data can support its efficacy and safety profile.

#### 3.1.5. Leronlimab

Leronlimab is a monoclonal antibody against CCR5, inhibiting the entry of SARS-CoV-2 into cells. CCR5 receptors are located on several antigen-presenting cells (e.g., T cells, dendritic cells, macrophages, and Langerhans cells) [74]. On March 31st, 2020, the FDA gave clearance for the initiation of a phase II trial evaluating leronlimab in patients with mild to moderate respiratory complications. However, multiple further studies to assess the efficacy, and benefits are still needed.

### 3.2. Antivirals

#### 3.2.1. Remdesivir

Developed during the Ebola virus outbreak in 2016, remdesivir is a promising therapy in treating COVID-19 [29, 70, 75]. It is a broad-spectrum antiviral agent. It prevents viral replication by inhibiting RNA-dependent RNA polymerase [2]. Remdesivir has also been used to treat SARS, MERS-CoV, and other viral illnesses. Remdesivir has a superior anti-MERS activity compared with lopinavir and ritonavir both in vitro and in vivo and displayed anti-SARS-CoV-2 ability in vitro [7, 69]. A randomized, placebo-controlled, clinical trial of 1059 COVID-19 patients revealed a median recovery time of 11 days (95% confidence interval [CI], 9–12) versus 15 days (95% CI, 13–19) in those who received placebo (rate ratio for recovery, 1.32; 95% CI, 1.12 to 1.55; *P* < 0.001). However, there was no significant difference in mortality (7.1% vs. 11.9% hazard ratio for death, 0.70; 95% CI, 0.47–1.04) [76]. A randomized, double-blind, placebo-controlled, multicenter, phase-III trial was conducted in China to evaluate the efficacy of remdesivir [75]. The study included 237 severely ill COVID-19 patients among whom 158 received remdesivir and 79 received placebo. The primary endpoint was time to clinical improvement [7, 77]. Patients receiving remdesivir had a faster time to clinical improvement than those receiving placebo, but this was not statistically significant (hazard ratio, 1·52 [0·95–2·43]). In the USA, compassionate use of remdesivir displayed clinical improvement in 36 of 53 hospitalized severe COVID-19 patients and 14 of 17 patients in an infectious disease ward [78, 79]. In meta-analysis with four studies, remdesivir was found to reduce 14 days mortality (OR, 0.61, CI 0.41–0.91) and need for mechanical ventilation (OR, 0.73; CI, 0.54–0.97) [80]. Remdesivir was given an “emergency use authorization” approval by FDA for patients with severe COVID-19 on May 1st, 2020 [81].

#### 3.2.2. Lopinavir/Ritonavir

Lopinavir/ritonavir is a combination antiretroviral drug that has been used to treat HIV and has also been part of clinical trials in the treatment of MERS-CoV and SARS-CoV-2. Lopinavir, an HIV-1 protease inhibitor, prevents viral gag-pol polyprotein cleavage, which results in immature, noninfectious viral particles; it was found to have activity against SARS-CoV-2 when used with ritonavir, a P450 CPY3A inhibitor, which increases plasma levels of lopinavir [82–85]. It has been widely used for the COVID-19 treatment in South Korea and Thailand. However, there is limited data on the efficacy and safety among COVID-19 patients [86–88]. Cao et al. found no benefit with lopinavir-ritonavir treatment in 199 hospitalized patients with severe COVID-19, demonstrating similar mortality rates (19.2%) than the standard of care group (25%) [38]. A single-center, controlled trial reached a similar conclusion for hospitalized patients with mild to moderate COVID-19 as compared with standard treatment [89]. In another study by Cheng et al., lopinavir/ritonavir did not shorten the duration of SARS-CoV-2 shedding [89, 90]. Gastrointestinal disturbance was the most common adverse effect associated with lopinavir-ritonavir, seen in 28% of patients. Moreover, the use of lopinavir/ritonavir was also associated with liver damage in COVID-19 [70, 91]. However, when used in combination with interferon-*β*-1b and ribavirin, lopinavir/ritonavir was found to be effective and superior to lopinavir/ritonavir alone and was associated with clinical improvement and shorter duration of hospital stay [92].

#### 3.2.3. Ribavirin

Ribavirin acts by inhibiting viral RNA-dependent RNA polymerase. Evidence regarding ribavirin use in COVID-19 patients has revealed no definitive role in some studies [27]. However, based on the data available, combination therapy with interferon-*α* or lopinavir-ritonavir may provide some clinical efficacy [29, 54, 70, 92, 93]. However, dose-dependent adverse events like liver and hematologic toxicities were the limiting factors [29]. Therefore, its role in treating COVID-19 patients remains inconclusive due to limited data and research.

#### 3.2.4. Favipiravir

Favipiravir is an inhibitor of RNA-dependent RNA polymerase approved for treating influenza, Ebola, and norovirus [58, 70, 94, 95]. Data from preliminary studies revealed significant clinical benefits (i.e., more rapid viral clearance of 4 days vs. 11 days) and improved chest imaging in COVID-19 patients than lopinavir-ritonavir alone (91.4% vs. 62.2%). In addition, there were fewer adverse events in patients administered favipiravir compared with those taking lopinavir-ritonavir alone (11.4% versus 55.6%) [96]. Another prospective, randomized, clinical trial reported better control of fever, cough, and respiratory symptoms among COVID-19 patients, with an improved recovery rate in patients receiving favipiravir compared with umifenovir (71.4% versus 55.9%) [70, 97]. However, these studies were limited by only evaluating noncritically ill patients.

#### 3.2.5. Oseltamivir

Oseltamivir is a neuraminidase inhibitor long used for the treatment of influenza A and B [29, 54]. However, current evidence has failed to find any efficacy and benefit for the treatment of COVID-19 patients, and hence, its use in any form should be discouraged [92].

#### 3.2.6. Umifenovir

Umifenovir is a membrane fusion inhibitor, preventing the interaction between viral S-proteins and ACE2 receptors [70]. It has been used as prophylaxis and for the treatment of influenza A and B [54, 97]. Apart from its benefit for influenza, umifenovir has shown antiviral effects for other viruses like hepatitis C virus, hepatitis B virus, Ebola virus, human herpesvirus 8, Lassa virus, and poliovirus [95]. A retrospective cohort study of 16 COVID-19 patients after 14 days of umifenovir and lopinavir-ritonavir administration versus lopinavir-ritonavir treatment alone showed 4% clearance of SARS-CoV-2 in the umifenovir experimental group compared with 53% patients in the control group [98]. Furthermore, there was an improvement in the chest CT scans in the experimental group (69% compared to 29% in lopinavir-ritonavir monotherapy) [98]. In another similar study of 16 patients taking umifenovir (200 mg TID), there was complete clearance of the virus versus 44.1% viral load detection in patients taking lopinavir-ritonavir monotherapy (400 mg/100 mg BID) [99]. However, other studies have found benefits limited to only nonseverely ill patients [100]. Due to the limited data available, its utilization is not routinely recommended at this time.

### 3.3. IV Immunoglobulins

The data regarding intravenous immunoglobulin (IVIG) efficacy and outcomes in COVID-19 patients is scarce. However, IVIG may help severe SARS-CoV-2 infection through immune modulation by saturating the Fc*γ*R [101]. In a case series of three critically ill SARS-CoV-2 patients in China who received IVIG at 0.3–0.4 g/kg/day for five days, all respiratory symptoms showed significant improvements in clinical status, and there were alleviated side effects [102]. However, a clear demonstration of therapeutic benefit will require further studies.

### 3.4. Convalescent Plasma

Treatment strategies for critically ill COVID-19 patients continue to be hampered by limited evidence. Administration of convalescent plasma (CP) has been long practiced to improve the survival rate during viral outbreaks, including SARS-CoV, MERS-CoV, and Ebola virus [70, 103–105]. A meta-analysis reported a significant reduction in mortality and viral load with CP immunotherapy, leading to the August 23rd, 2020, FDA decision to authorize emergency use in the treatment of COVID-19 [106]. The role of convalescent plasma transfusion in COVID-19 patients is related to both an increase in viral clearance and inhibition of the cytokine storm that plays a major role in precipitating organ damage [107]. Earlier in the current pandemic, small observational studies revealed promising outcomes and clinical improvements from the CP utilization [108–111]. The first study from Wuhan reported outcomes of 5 critically ill patients who were administered CP [109]. In four of the five patients, there was remarkable improvement in the clinical status measured by the Alveolar-arterial (A/a) gradient and chest CT scan, and there was a decrease in inflammatory biomarkers, as well [109]. Duan et al. reported that after a single transfusion of convalescent plasma in 10 patients, there were no adverse events [108]. Additionally, similar clinical improvements were reported in other small case studies [109, 111].

The timing of infusion appears to play a role in the success of the treatment. Early data appears to support the fact that transfusions done earlier in the disease proved to have better outcomes, including mortality [106, 112, 113]. Zeng et al. reported poor mortality outcomes when convalescent plasma was administered late [114]. A multicenter cohort study consisting of 35,322 patients reported a seven-day mortality rate of 8.7% [95% CI, 8.3%–9.2%] when plasma was transfused within three days of COVID-19 diagnosis compared with 11.9% [11.4%–12.2%] (*P* < 0.001) when the transfusion was done after three days of diagnosis. In addition, there was a significant reduction in 30-day mortality in early transfused patients (21.6% vs. 26.7%; *P* < 0.0001) (NCT04338360). Moreover, in an RCT of 86 patients, the patients were symptomatic for ten days, 79% of tested patients had COVID-19 neutralizing antibodies comparable to median titers with those of donors. As patients developed neutralizing antibodies as early as the first week, convalescent plasma is likely to help only patients with recent clinical symptoms [115]. In another randomized control trial of 103 patients with severe or life-threatening COVID-19 disease, convalescent plasma did not provide any benefit in terms of 28 days mortality (OR, 0.59; 95% CI, 0.22–1.59; *P*=0.30) or in terms of time from randomization to discharge(HR, 1.61; 95% CI, 0.88–2.95; *P*=0.12) [116]. A randomized controlled trial that enrolled 334 patients to compare convalescent plasma with placebo could not find any difference in terms of clinical outcomes and mortality. The median days of patient randomized after appearance of symptoms was 8 days [117].

The use of plasma transfusion therapy may be an option for critically ill patients if administered early in the disease course. However, many factors, including the number of transfusions, adjustments based on body mass index, donor antibody titers, and other parameters, need to be evaluated to optimize this therapy.

### 3.5. Miscellaneous

#### 3.5.1. Angiotensin-Converting Enzyme (ACE) Inhibitors

The SARS-CoV-2 interacts with the ACE2 receptor to enter the host cell. Inhibiting this step can be a potential target for COVID-19 treatment [118]. German guidelines recommended the compassionate use of ACE-II inhibitors [119]. However, many clinical experts have discouraged their usage. They have postulated that blocking ACE-II receptors with ACE inhibitors may lead to poorer outcomes due to the upregulation of the receptors and thus increasing viral entry into the host cells [120]. However, others have challenged this position because receptor upregulation appears to seldom occur at therapeutic doses [121]. Therefore, it is currently recommended that patients with cardiovascular comorbidities continue to take ACE inhibitors and ARBs as prescribed [122].

#### 3.5.2. Azithromycin

Azithromycin (AZT), a frequently used antibiotic, has been used in combination therapy with HCQ for COVID-19 patients. A multicenter retrospective study of 1438 hospitalized patients evaluated the efficacy and side effects of HCQ plus AZT combination therapy, compared with HCQ alone, AZT alone, and a placebo control group, and found no significant differences in the experimental groups to the control group [18]. Furthermore, combination therapy was associated with cardiac arrest (OR = 2.13) [18]. Magagnoli et al. reported similar results that combination therapy with HCQ plus AZT did not decrease the risk of death in COVID-19 patients [17].

Additionally, in patients who were only taking HCQ, the risk of death was even higher [17]. Due to the adverse cardiovascular side effects with HCQ and AZT combination therapy, this combination's clinical use requires frequent ECG monitoring [26, 27]. Based on the above data, routine usage cannot be recommended at this time.

#### 3.5.3. Anticoagulation (AC)

One of the major complications associated with hospitalized COVID-19 patients is life-threatening thromboembolic events. Abnormal coagulation in COVID-19 patients is independently associated with an increased risk of mortality [123]. Therefore, identifying high-risk patients and early institutions of antithrombotic is essential to limit thrombus formation and treat systemic thromboembolic complications in COVID-19 patients. The International Society on Thrombosis and Hemostasis has recommended antithrombotic prophylaxis with low-molecular-weight heparin (LMWH) for all hospitalized patients, except contraindicated [123]. However, routine thromboprophylaxis is not preferred in ambulatory patients with active medical illness [124]. Apart from its anticoagulation effects, unfractionated heparin has demonstrated antiviral properties in some studies [125].

Unfractionated heparin is frequently preferred in patients with underlying renal disease [125]. However, the choice of AC should be individualized based on patient clinical status, as many patients might progress from a thrombotic state to a bleeding pattern due to platelet destruction and coagulation factor consumption [122,123]. In obese patients with BMI ≥40 kg/m^2^, a higher dose of thromboprophylaxis has been shown to decrease VTE risk by 50% [126].

Aggressive thromboprophylaxis with high doses of anticoagulation can be considered in patients who meet the high sepsis-induced coagulopathy (SIC) score criteria or in patients with markedly elevated D-dimer levels [123]. Tang et al. found that aggressive thromboprophylaxis was associated with better outcomes in a study of 449 COVID-19-positive patients [127]. Moreover, in patients on low-molecular-weight heparin (LMWH) with SIC score ≥ four or D-dimer ≥ 6 times, the upper limit had a significantly lower 28-day mortality rate than untreated patients (40.0% vs 64.2%; *P*=0.029) [127].

Few studies have reported improved survival benefits with fibrinolytic therapy in patients with acute lung injury and ARDS [128, 129]. However, in a case series of 3 COVID-19 patients administered tPA (alteplase) suffering from ARDS and respiratory failure, there was an initial, but only transient, improvement in the PaO_2_/FiO_2_ ratio [129]. Therefore, more studies are required to evaluate outcomes associated with plasminogen activator in COVID-19 patients [129].

## 4. Conclusion

The treatment of COVID-19 remains an immense challenge worldwide due to limited evidence and rapidly evolving and changing treatment options. The treatment repurposed and showing promising results should be tailored based on clinical conditions and clinical expertise. The ongoing research/trials investigate the safety and efficacy of various drug options.

## Figures and Tables

**Table 1:** Narrative summary of the commonly used therapeutic agents for COVID-19.

Drug	MOA	Dosage	Duration	COVID-specific indication	Adverse effects	Results	Caution	Evidence
Hydroxychloroquine	Unclear; increases intracellular pH to inhibit lysosomal activity in APC and B cells to prevent T-cell and cytokine activation	600 mg PO BID × 2 days, then 400 mg qd × 4 days	6 days	(i) US FDA emergency use authorized for hospitalized patients (ii) Possible postexposure prophylaxis	(i) Oculotoxicity (ii) Worsening of skin disease (iii) Cardiotoxicity (iv) Arrhythmia		(i) Renal and hepatic impairment is not defined (ii) Psoriasis (iii) G6PD deficiency (iv) QT prolongation (v) Arrythmia/CHF	(i) No benefit
Chloroquine	Inhibit early stages of final virus replication	(i) High dose: 600 mg BID × 10 days (ii) Low dose: 450 mg BID × 1 day, then 450 mg qd × 4 days	5–10 days	US FDA emergency use authorized for hospitalized patients			(i) Renal: CrCl <10 decrease 50% dose (ii) Hepatic: not defined (iii) Seizure history (iv) Psoriasis (v) G6PD deficiency (vi) QT prolongation (vii) Arrythmia/CHF	(i) No benefit
Tocilizumab	IgG1 IL-6 receptor antibody	400 mg	Single dose	US FDA approved for phase III clinical trial in hospitalized patients with severe infection			(i) Renal: CrCL < 30 not defined (ii) Hepatic: avoid use in active hepatic disease or baseline AST/ALT >1.5 ULN	(i) Benefit in specified group
Dexamethasone	Unclear; anti-inflammatory			WHO recommends for hospitalized COVID-19	(i) Increased blood glucose (ii) Psychosis (iii) Avascular osteonecrosis		(i) Hepatic: not defined (ii) Osteoporosis (iii) Psychiatric disorder (iv) Diabetes mellitus (v) Hypertension (vi) 1^st^ trimester pregnancy (vii) Immunosuppressed	(i) Benefit in moderate to severe cases
Leronlimab	CCR5 receptor antibody to prevent viral entry into cells	700 mg SC weekly		US FDA approved for phase II trials in patients with mild to moderate respiratory complications				
Lopinavir/ritonavir	Lopinavir: HIV-1 protease inhibitor Ritonavir: P450 CPY3A inhibitor to increase lopinavir levels	400/100 mg PO BID	14 days		(i) Pancreatitis (ii) Diarrhea (iii) Abdominal pain (iv) Hgb drop >2 g/dL (v) Liver dysfunction (vi) 2.3% mortality noted in cases with delayed administration		(i) Hepatic: not defined (ii) QT prolongation (iii) History of pancreatitis (iv) Diabetes mellitus (v) Ischemic heart disease (vi) Myasthenia gravis	Mixed data regarding efficacy in viral loads
Remdesivir	RNA-dependent RNA polymerase adenosine nucleotide analog	Day 1 200 mg IV, followed by 100 mg IV QD	10 days	Several trials			(i) Renal: not defined in eGFR<30 (ii) Hepatic: not defined, avoid in ALT>5x ULN	(i) SARS MERS, Ebola use indicated antiviral activity in vitro (ii) Mixed findings
Azithromycin	Unclear for indirect immunomodulation activity	500 mg × 1 day, then 250 mg qd × 4 days	5 days	In COVID-19, used for immunomodulating on role rather than antibiotic benefit.	(i) QT prolongation		(i) Renal: CrCl<10 not defined (ii) Hepatic: not defined (iii) QT prolongation (iv) Ventricular arrythmia (v) CHF (vi) Myasthenia gravis	(i) No benefit
Convalescent plasma IgG	Virus-specific neutralizing antibody promotes virus clearance by host; passive immunity	200–400 mL	Single transfusion		(i) Fever (ii) Chills (iii) Allergic reactions (iv) Bronchoconstriction (v) Volume overload			(i) Used during Ebola and MERS outbreaks (ii) Mixed findings

## Data Availability

This is a review article based on secondary data from other articles so we do not have our own data!

## References

[1] World Health Organization (2021). *WHO Coronavirus (COVID-19) Dashboard | WHO Coronavirus (COVID-19) Dashboard with Vaccination Data*.

[2] Kupferschmidt K. (2020). WHO launches global megatrial of the four most promising coronavirus treatments. *Science*.

[3] Food T. U. S. (2020). *Coronavirus (COVID-19) Update*.

[4] Wilson K. C., Chotirmall S. H., Bai C., Rello J. (2020). COVID-19: interim guidance on management pending empirical evidence. From an American Thoracic Society-led international task force on behalf of the international task force on COVID-19. http://www.thoracic.org.

[5] Savarino A., Boelaert J. R., Cassone A., Majori G., Cauda R. (2003). Effects of chloroquine on viral infections: an old drug against today’s diseases. *The Lancet Infectious Diseases*.

[6] Cortegiani A., Ingoglia G., Ippolito M., Giarratano A., Einav S. (2020). A systematic review on the efficacy and safety of chloroquine for the treatment of COVID-19. *Journal of Critical Care*.

[7] Wang M., Cao R., Zhang L. (2020). Remdesivir and chloroquine effectively inhibit the recently emerged novel coronavirus (2019-nCoV) in vitro. *Cell Research*.

[8] An J., Minie M., Sasaki T., Woodward J. J., Elkon K. B. (2017). Antimalarial drugs as immune modulators: new mechanisms for old drugs. *Annual Review of Medicine*.

[9] van den Borne B. E. E. M., Dijkmans B. A. C., de Rooij H. H., le Cessie S., Verweij C. L. (1997). Chloroquine and hydroxychloroquine equally affect tumor necrosis factor-*α*, interleukin 6, and interferon-*γ* production by peripheral blood mononuclear cells. *Journal of Rheumatology*.

[10] Wallace D. J., Linker-Israeli M., Hyun S., Klinenberg J. R., Stecher V. (1994). The effect of hydroxychloroquine therapy on serum levels of immunoregulatory molecules in patients with systemic lupus erythematosus. *Journal of Rheumatology*.

[11] Gao J., Tian Z., Breakthrough Y. X. (2020). Chloroquine phosphate has shown apparent efficacy in treatment of COVID-19 associated pneumonia in clinical studies. *BioScience Trends*.

[12] Gautret P., Lagier J.-C., Parola P. (2020). Hydroxychloroquine and azithromycin as a treatment of COVID-19: results of an open-label non-randomized clinical trial. *International Journal of Antimicrobial Agents*.

[13] Gautret P., Lagier J. C., Parola P. (2020). Clinical and microbiological effect of a combination of hydroxychloroquine and azithromycin in 80 COVID-19 patients with at least a six-day follow up: a pilot observational study. *Travel Medicine and Infectious Disease*.

[14] Chen J., Liu D., Liu L. (2020). A pilot study of hydroxychloroquine in treatment of patients with moderate COVID-19. *Zhejiang da xue xue bao Yi xue ban = Journal of Zhejiang University Medical Sciences*.

[15] Geleris J., Sun Y., Platt J. (2020). Observational study of hydroxychloroquine in hospitalized patients with covid-19. *New England Journal of Medicine*.

[16] Arshad S., Kilgore P., Chaudhry Z. S., Jacobsen G., Wang D. D., Huitsing K. (2020). Treatment with hydroxychloroquine, azithromycin, and combination in patients hospitalized with COVID-19. *International Journal of Infectious Diseases*.

[17] Magagnoli J., Narendran S., Pereira F. (2020). Outcomes of hydroxychloroquine usage in United States veterans hospitalized with Covid-19. *medRxiv*.

[18] Rosenberg E. S., Dufort E. M., Udo T. (2020). Association of treatment with hydroxychloroquine or azithromycin with in-hospital mortality in patients with COVID-19 in New York state. *Jama*.

[19] Cavalcanti A. B., Zampieri F. G., Rosa R. G. (2020). Hydroxychloroquine with or without azithromycin in mild-to-moderate covid-19. *New England Journal of Medicine*.

[20] Tang W., Cao Z., Han M. (2020). Hydroxychloroquine in patients with mainly mild to moderate coronavirus disease 2019: open label, randomised controlled trial. *BMJ*.

[21] Skipper C. P., Pastick K. A., Engen N. W. (2020). Hydroxychloroquine in nonhospitalized adults with early COVID-19 a randomized trial. *Annals of Internal Medicine*.

[22] Mitjà O., Corbacho-Monné M., Ubals M., Tebé C., Peñafiel J., Tobias A. (2020). Hydroxychloroquine for early treatment of adults with mild coronavirus disease 2019: a randomized, controlled trial. *Clinical Infectious Diseases*.

[23] Boulware D. R., Pullen M. F., Bangdiwala A. S. (2020). A randomized trial of hydroxychloroquine as postexposure prophylaxis for covid-19. *New England Journal of Medicine*.

[24] Chatterjee P., Anand T., Singh K. (2020). Healthcare workers & SARS-CoV-2 infection in India: a case-control investigation in the time of COVID-19. *Indian Journal of Medical Research*.

[25] Borsini F., Crumb W., Pace S. (2012). In VitroCardiovascular effects of dihydroartemisin-piperaquine combination compared with other antimalarials. *Antimicrobial Agents and Chemotherapy*.

[26] Asensio E., Acunzo R., Uribe W., Saad E. B., Sáenz L. C. (2020). Recommendations for the measurement of the QT interval during the use of drugs for COVID-19 infection treatment. Updatable in accordance with the availability of new evidence. *Journal of Interventional Cardiac Electrophysiology*.

[27] Bhimraj A., Morgan R. L., Shumaker A. H. (2020). Infectious diseases society of America guidelines on the treatment and management of patients with COVID-19. *Clinical Infectious Diseases: An Official Publication of the Infectious Diseases Society of America*.

[28] Kalil A. C. (2020). Treating COVID-19-off-label drug use, compassionate use, and randomized clinical trials during pandemics. *Jama*.

[29] Sanders J. M., Monogue M. L., Jodlowski T. Z., Cutrell J. B. (2020). Pharmacologic treatments for coronavirus disease 2019 (COVID-19): a review. *JAMA - Journal of the American Medical Association. American Medical Association*.

[30] Chorin E., Dai M., Shulman E. (2020). The QT interval in patients with SARS-CoV-2 infection treated with hydroxychloroquine/azithromycin. *medRxiv*.

[31] Mercuro N. J., Yen C. F., Shim D. J. (2020). Risk of QT interval prolongation associated with use of hydroxychloroquine with or without concomitant azithromycin among hospitalized patients testing positive for coronavirus disease 2019 (COVID-19). *JAMA Cardiology*.

[32] Silva Borba M. G., Almeida Val F. F., Sampaio V. S., Araújo Alexandre M. A., Melo G. C., Brito M. (2020). Chloroquine diphosphate in two different dosages as adjunctive therapy of hospitalized patients with severe respiratory syndrome in the context of coronavirus (SARS-CoV-2) infection: preliminary safety results of a randomized, double-blinded, phase IIb clinical trial (CloroCovid-19 Study). *JAMA Network Open*.

[33] Bessière F., Roccia H., Delinière A. (2020). Assessment of QT intervals in a case series of patients with coronavirus disease 2019 (COVID-19) infection treated with hydroxychloroquine alone or in combination with azithromycin in an intensive care unit. *JAMA Cardiology*.

[34] Jorge A., Ung C., Young L. H., Melles R. B., Choi H. K. (2018). Hydroxychloroquine retinopathy - implications of research advances for rheumatology care. *Nature Reviews Rheumatology*.

[35] Ray W. A., Murray K. T., Hall K., Arbogast P. G., Stein C. M. (2012). Azithromycin and the risk of cardiovascular death. *New England Journal of Medicine*.

[36] Lu Z. K., Yuan J., Li M. (2015). Cardiac risks associated with antibiotics: azithromycin and levofloxacin. *Expert Opinion on Drug Safety*.

[37] https://www.medicalnewstoday.com/articles/257624#1.

[38] Cao B., Wang Y., Wen D. (2020). A trial of lopinavir-ritonavir in adults hospitalized with severe covid-19. *New England Journal of Medicine*.

[39] Wang D., Hu B., Hu C. (2020). Clinical characteristics of 138 hospitalized patients with 2019 novel coronavirus-infected pneumonia in Wuhan, China. *Jama*.

[40] http://www.metaevidence.org/COVID19.aspx.

[41] Mehta P., McAuley D. F., Brown M., Sanchez E., Tattersall R. S., Manson J. J. (2020). COVID-19: consider cytokine storm syndromes and immunosuppression. *The Lancet*.

[42] Rhen T., Cidlowski J. A. (2005). Antiinflammatory action of glucocorticoids - new mechanisms for old drugs. *New England Journal of Medicine*.

[43] Horby P., Lim W. S., Emberson J., Mafham M., Bell J., Linsell L. (2021). Effect of dexamethasone in hospitalized patients with COVID-19 – preliminary report. *New England Journal of Medicine*.

[44] Wu J., Huang J., Zhu G. (2020). Systemic corticosteroids show no benefit in severe and critical COVID-19 patients in Wuhan, China: a retrospective cohort study. *medRxiv*.

[45] Lu X., Chen T., Wang Y., Wang J., Yan F. (2020). Adjuvant corticosteroid therapy for critically ill patients with COVID-19. *Critical Care*.

[46] System H. F. H. (2020). Early short course corticosteroids in COVID-19. ClinicalTrials.gov. https://europepmc.org/article/PPR/PPR158645.

[47] Wang Y., Jiang W., He Q. (2020). Early, low-dose and short-term application of corticosteroid treatment in patients with severe COVID-19 pneumonia: single-center experience from Wuhan, China. *medRxiv*.

[48] Chroboczek T., Lacoste M., Wackenheim C. (2020). Beneficial effect of corticosteroids in severe COVID-19 pneumonia: a propensity score matching analysis. *medRxiv*.

[49] Group T. R. C. (2021). Dexamethasone in hospitalized patients with covid-19. *New England Journal of Medicine*.

[50] Ling Y., Xu S.-B., Lin Y.-X. (2020). Persistence and clearance of viral RNA in 2019 novel coronavirus disease rehabilitation patients. *Chinese Medical Journal*.

[51] Russell C. D., Millar J. E., Baillie J. K. (2020). Clinical evidence does not support corticosteroid treatment for 2019-nCoV lung injury. *The Lancet*.

[52] Shang L., Zhao J., Hu Y., Du R., Cao B. (2020). On the use of corticosteroids for 2019-nCoV pneumonia. *The Lancet*.

[53] Zhou W., Liu Y., Tian D. (2020). Potential benefits of precise corticosteroids therapy for severe 2019-nCoV Pneumonia. *Signal Transduction and Targeted Therapy*.

[54] Yang Z., Liu J., Zhou Y., Zhao X., Zhao Q., Liu J. (2020). The effect of corticosteroid treatment on patients with coronavirus infection: a systematic review and meta-analysis. *Journal of Infection*.

[55] Fatima S. A., Asif M., Khan K. A., Siddique N., Khan A. Z. (2020). Comparison of efficacy of dexamethasone and methylprednisolone in moderate to severe covid 19 disease. *Annals of Medicine and Surgery*.

[56] Ranjbar K., Moghadami M., Mirahmadizadeh A. (2021). Methylprednisolone or dexamethasone, which one is superior corticosteroid in the treatment of hospitalized COVID-19 patients: a triple-blinded randomized controlled trial. *BMC Infectious Diseases*.

[57] Wu R., Wang L., Kuo H.-C. D. (2020). An update on current therapeutic drugs treating COVID-19. *Current Pharmacology Reports*.

[58] Ruan Q., Yang K., Wang W., Jiang L., Song J. (2020). Clinical predictors of mortality due to COVID-19 based on an analysis of data of 150 patients from Wuhan, China. *Intensive Care Medicine*.

[59] Luo P., Liu Y., Qiu L., Liu X., Liu D., Li J. (2020). Tocilizumab treatment in COVID‐19: a single center experience. *Journal of Medical Virology*.

[60] Xu X., Han M., Li T. (2020). Effective treatment of severe COVID-19 patients with tocilizumab. *Proceedings of the National Academy of Sciences*.

[61] Rosas I. O., Bräu N., Waters M., Go R. C., Hunter B. D., Bhagani S. (2021). Tocilizumab in hospitalized patients with severe covid-19 pneumonia. *New England Journal of Medicine*.

[62] Horby P. W., Mafham M., Bell J. L. (2020). Lopinavir–ritonavir in patients admitted to hospital with COVID-19 (RECOVERY): a randomised, controlled, open-label, platform trial. *The Lancet*.

[63] Haagmans B. L., Kuiken T., Martina B. E. (2004). Pegylated interferon-*α* protects type 1 pneumocytes against SARS coronavirus infection in macaques. *Nature Medicine*.

[64] Ghamdi M., Alghamdi K. M., Ghandoora Y. (2016). Treatment outcomes for patients with middle eastern respiratory syndrome coronavirus (MERS CoV) infection at a coronavirus referral center in the Kingdom of Saudi Arabia. *BMC Infectious Diseases*.

[65] Belhadi D., Peiffer-Smadja N., Lescure F. X., Yazdanpanah Y., Mentré F., Laouénan C. (2020). A brief review of antiviral drugs evaluated in registered clinical trials for COVID-19. *medRxiv*.

[66] Martinez M. A. (2020). Compounds with therapeutic potential against novel respiratory 2019 coronavirus. *Antimicrobial Agents and Chemotherapy*.

[67] Sallard E., Lescure F. X., Yazdanpanah Y., Mentre F., Peiffer-Smadja N. (2020). Type 1 interferons as a potential treatment against COVID-19. *Antiviral Research*.

[68] Arabi Y. M., Alothman A., Balkhy H. H. (2018). Treatment of Middle East Respiratory Syndrome with a combination of lopinavir-ritonavir and interferon-*β*1b (MIRACLE trial): study protocol for a randomized controlled trial. *Trials*.

[69] Sheahan T. P., Sims A. C., Leist S. R. (2020). Comparative therapeutic efficacy of remdesivir and combination lopinavir, ritonavir, and interferon beta against MERS-CoV. *Nature Communications*.

[70] Lam S., Lombardi A., Ouanounou A. (2020). COVID-19: a review of the proposed pharmacological treatments. *European Journal of Pharmacology*.

[71] Chan J. F. W., Chan K.-H., Kao R. Y. T. (2013). Broad-spectrum antivirals for the emerging Middle East respiratory syndrome coronavirus. *Journal of Infection*.

[72] Dong L., Hu S., Gao J. (2020). Discovering drugs to treat coronavirus disease 2019 (COVID-19). *Drug Discoveries & Therapeutics*.

[73] Lokugamage K., Hage A., de Vries M. (2020). Type I interferon susceptibility distinguishes SARS-CoV-2 from SARS-CoV. *Journal of Virology*.

[74] Pattterson B., Seetthamraju H., Dhody K., Corley M., Kazempour K., Lalezari J. (2020). Disruption of the CCL5/RANTES-CCR5 pathway restores immune homeostasis and reduces plasma viral load in critical COVID-19. *medRxiv: The Preprint Server for Health Sciences*.

[75] Ko W. C., Rolain J. M., Lee N. Y. (2020). Arguments in favour of remdesivir for treating SARS-CoV-2 infections. *International Journal of Antimicrobial Agents*.

[76] Beigel J. H., Tomashek K. M., Dodd L. E. (2020). Remdesivir for the treatment of covid-19 - final report. *New England Journal of Medicine*.

[77] Wang Y., Zhang D., Du G. (2020). Remdesivir in adults with severe COVID-19: a randomised, double-blind, placebo-controlled, multicentre trial. *The Lancet*.

[78] Grein J., Ohmagari N., Shin D. (2020). Compassionate use of remdesivir for patients with severe covid-19. *New England Journal of Medicine*.

[79] Antinori S., Cossu M. V., Ridolfo A. L., Rech R., Bonazzetti C., Pagani G. (2020). Compassionate remdesivir treatment of severe Covid-19 pneumonia in intensive care unit (ICU) and Non-ICU patients: clinical outcome and differences in post-treatment hospitalisation status. *Pharmacological Research*.

[80] Shrestha D. B., Budhathoki P., Syed N.-i.-H., Rawal E., Raut S., Khadka S. (2021). Remdesivir: a potential game-changer or just a myth? A systematic review and meta-analysis. *Life Sciences*.

[81] https://www.fda.gov/emergency-preparedness-and-response/mcm-legal-regulatory-and-policy-framework/emergency-use-authorization.

[82] Chan K. S., Lai S. T., Chu C. M., Tsui E., Tam C. Y., Wong M. M. L. (2003). Treatment of severe acute respiratory syndrome with lopinavir/ritonavir: a multicentre retrospective matched cohort study. *Hong Kong Medical Journal*.

[83] Chu C. M., Cheng V. C. C., Hung I. F. N. (2004). Role of lopinavir/ritonavir in the treatment of SARS: initial virological and clinical findings. *Thorax*.

[84] Chan J. F.-W., Yao Y., Yeung M.-L. (2015). Treatment with lopinavir/ritonavir or interferon-*β*1b improves outcome of MERS-CoV infection in a nonhuman primate model of common marmoset. *Journal of Infectious Diseases*.

[85] Zumla A., Chan J. F. W., Azhar E. I., Hui D. S. C., Yuen K.-Y. (2016). Coronaviruses - drug discovery and therapeutic options. *Nature Reviews Drug Discovery*.

[86] Lim J., Jeon S., Shin H. Y., Kim M. J., Seong Y. M., Lee W. J. (2020). Case of the index patient who caused tertiary transmission of coronavirus disease 2019 in Korea: the application of lopinavir/ritonavir for the treatment of COVID-19 pneumonia monitored by quantitative RT-PCR. *Journal of Korean Medical Science*.

[87] Kim J. Y. (2020). Letter to the editor: case of the index patient who caused tertiary transmission of coronavirus disease 2019 in Korea: the application of lopinavir/ritonavir for the treatment of COVID-19 pneumonia monitored by quantitative RT-PCR. *Journal of Korean Medical Science*.

[88] Chen J., Ling Y., Xi X., Liu P., Li F., Li T. (2020). Efficacies of lopinavir/ritonavir and abidol in the treatment of novel coronavirus pneumonia. *Chinese Journal of Infectious Diseases*.

[89] Li Y., Xie Z., Lin W., Cai W., Wen C., Guan Y. (2020). An exploratory randomized controlled study on the efficacy and safety of lopinavir/ritonavir or arbidol treating adult patients hospitalized with mild/moderate COVID-19 (ELACOI). *medRxiv*.

[90] Cheng C.-Y., Lee Y.-L., Chen C.-P. (2020). Lopinavir/ritonavir did not shorten the duration of SARS CoV-2 shedding in patients with mild pneumonia in Taiwan. *Journal of Microbiology, Immunology, and Infection*.

[91] Fan Z., Chen L., Li J. (2020). Clinical features of COVID-19-related liver damage. *SSRN Electronic Journal*.

[92] Hung I. F.-N., Lung K.-C., Tso E. Y.-K. (2020). Triple combination of interferon beta-1b, lopinavir-ritonavir, and ribavirin in the treatment of patients admitted to hospital with COVID-19: an open-label, randomised, phase 2 trial. *The Lancet*.

[93] Yousefi B., Valizadeh S., Ghaffari H., Vahedi A., Karbalaei M., Eslami M. (2020). A global treatments for coronaviruses including COVID‐19. *Journal of Cellular Physiology*.

[94] Coomes E. A., Haghbayan H. (2020). Favipiravir, an antiviral for COVID-19?. *Journal of Antimicrobial Chemotherapy*.

[95] McKee D. L., Sternberg A., Stange U., Laufer S., Naujokat C. (2020). Candidate drugs against SARS-CoV-2 and COVID-19. *Pharmacological Research*.

[96] Cai Q., Yang M., Liu D. (2020). Experimental treatment with favipiravir for COVID-19: an open-label control study. *Engineering*.

[97] Chen C., Zhang Y., Huang J., Yin P., Cheng Z., Wu J. (2020). Favipiravir versus Arbidol for COVID-19: a randomized clinical trial. *medRxiv*.

[98] Deng L., Li C., Zeng Q. (2020). Arbidol combined with LPV/r versus LPV/r alone against Corona Virus Disease 2019: a retrospective cohort study. *Journal of Infection*.

[99] Zhu Z., Lu Z., Xu T. (2020). Arbidol monotherapy is superior to lopinavir/ritonavir in treating COVID-19. *Journal of Infection*.

[100] Lian N., Xie H., Lin S., Huang J., Zhao J., Lin Q. (2020). Umifenovir treatment is not associated with improved outcomes in patients with coronavirus disease 2019: a retrospective study. *Clinical Microbiology and Infections*.

[101] Schwab I., Nimmerjahn F. (2013). Intravenous immunoglobulin therapy: how does IgG modulate the immune system?. *Nature Reviews Immunology*.

[102] Cao W., Liu X., Bai T. (2020). High-dose intravenous immunoglobulin as a therapeutic option for deteriorating patients with coronavirus disease 2019. *Open Forum Infectious Diseases*.

[103] Rajendran K., Krishnasamy N., Rangarajan J., Rathinam J., Natarajan M., Ramachandran A. (2020). Convalescent plasma transfusion for the treatment of COVID‐19: systematic review. *Journal of Medical Virology*.

[104] Rojas M., Rodríguez Y., Monsalve D. M. (2020). Convalescent plasma in Covid-19: possible mechanisms of action. *Autoimmunity Reviews*.

[105] Zhao Q., He Y. (2020). Challenges of convalescent plasma therapy on COVID-19. *Journal of Clinical Virology*.

[106] Mair-Jenkins J., Saavedra-Campos M., Baillie J. K. (2015). The effectiveness of convalescent plasma and hyperimmune immunoglobulin for the treatment of severe acute respiratory infections of viral etiology: a systematic review and exploratory meta-analysis. *Journal of Infectious Diseases*.

[107] Brown B. L., McCullough J. (2020). Treatment for emerging viruses: convalescent plasma and COVID-19. *Transfusion and Apheresis Science Official Journal of the World Apheresis Association:Official Journal of the European Society for Haemapheresis*.

[108] Duan K., Liu B., Li C. (2020). Effectiveness of convalescent plasma therapy in severe COVID-19 patients. *Proceedings of the National Academy of Sciences*.

[109] Shen C., Wang Z., Zhao F. (2020). Treatment of 5 critically ill patients with COVID-19 with convalescent plasma. *Jama*.

[110] Ye M., Fu D., Ren Y. (2020). Treatment with convalescent plasma for COVID‐19 patients in Wuhan, China. *Journal of Medical Virology*.

[111] Zhang B., Liu S., Tan T. (2020). Treatment with convalescent plasma for critically ill patients with severe acute respiratory syndrome coronavirus 2 infection. *Chest*.

[112] Soo Y. O. Y., Cheng Y., Wong R. (2004). Retrospective comparison of convalescent plasma with continuing high-dose methylprednisolone treatment in SARS patients. *Clinical Microbiology and Infections*.

[113] Cheng Y., Wong R., Soo Y. O. Y. (2004). Use of convalescent plasma therapy in SARS patients in Hong Kong. *European Journal of Clinical Microbiology & Infectious Diseases*.

[114] Zeng Q.-L., Yu Z.-J., Gou J.-J. (2020). Effect of convalescent plasma therapy on viral shedding and survival in patients with coronavirus disease 2019. *The Journal of Infectious Diseases*.

[115] Gharbharan A., Jordans C. C. E., Geurtsvankessel C., Hollander J. G., Karim F., Mollema F. P. N. (2020). Convalescent Plasma for COVID-19. A randomized clinical trial. *medRxiv*.

[116] Li L., Zhang W., Hu Y., Tong X., Zheng S., Yang J. (2020). Effect of convalescent plasma therapy on time to clinical improvement in patients with severe and life-threatening COVID-19: a randomized clinical trial. *Journal of the American Medical Association*.

[117] Simonovich V. A., Pratx L. D. B., Scibona P., Beruto M. v., Vallone M. G., Vázquez C. (2020). A randomized trial of convalescent plasma in covid-19 severe pneumonia.

[118] Hoffmann M., Kleine-Weber H., Schroeder S. (2020). SARS-CoV-2 cell entry depends on ACE2 and TMPRSS2 and is blocked by a clinically proven protease inhibitor. *Cell*.

[119] Kluge S., Janssens U., Welte T. (2020). Empfehlungen zur intensivmedizinischen Therapie von Patienten mit COVID-19. *Medizinische Klinik-Intensivmedizin und Notfallmedizin*.

[120] Esler M., Esler D. (2020). Can angiotensin receptor-blocking drugs perhaps be harmful in the COVID-19 pandemic?. *Journal of Hypertension*.

[121] Sriram K., Insel P. A. (2020). Risks of ACE inhibitor and ARB usage in COVID‐19: evaluating the evidence. *Clinical Pharmacology & Therapeutics*.

[122] Gurwitz D. (2020). Angiotensin receptor blockers as tentative SARS‐CoV‐2 therapeutics. *Drug Development Research*.

[123] Thachil J., Tang N., Gando S. (2020). ISTH interim guidance on recognition and management of coagulopathy in COVID-19. *Journal of Thrombosis and Haemostasis*.

[124] Lodigiani C., Iapichino G., Carenzo L. (2020). Venous and arterial thromboembolic complications in COVID-19 patients admitted to an academic hospital in Milan, Italy. *Thrombosis Research*.

[125] Barrett C. D., Moore H. B., Yaffe M. B., Moore E. E. (2020). ISTH interim guidance on recognition and management of coagulopathy in COVID-19: a comment. *Journal of Thrombosis and Haemostasis*.

[126] Wang T.-F., Milligan P. E., Wong C. A., Deal E. N., Thoelke M. S., Gage B. F. (2017). Efficacy and safety of high-dose thromboprophylaxis in morbidly obese inpatients. *Thrombosis & Haemostasis*.

[127] Tang N., Bai H., Chen X., Gong J., Li D., Sun Z. (2020). Anticoagulant treatment is associated with decreased mortality in severe coronavirus disease 2019 patients with coagulopathy. *Journal of Thrombosis and Haemostasis*.

[128] Hardaway R. M., Williams C. H., Marvasti M. (1990). Prevention of adult respiratory distress syndrome with plasminogen activator in pigs. *Critical Care Medicine*.

[129] Wang J., Hajizadeh N., Moore E. E. (2020). Tissue plasminogen activator (tPA) treatment for COVID-19 associated acute respiratory distress syndrome (ARDS): a case series. *Journal of Thrombosis and Haemostasis*.

